# Temporal changes in genetic diversity of *msp*-*1*, *msp*-*2*, and *msp*-*3* in *Plasmodium falciparum* isolates from Grande Comore Island after introduction of ACT

**DOI:** 10.1186/s12936-018-2227-3

**Published:** 2018-02-20

**Authors:** Bo Huang, Fei Tuo, Yuan Liang, Wanting Wu, Guangchao Wu, Shiguang Huang, Qirun Zhong, Xin-zhuan Su, Hongying Zhang, Mingqiang Li, Affane Bacar, Kamal Said Abdallah, Ahamada M. S. A. Mliva, Qi Wang, Zhaoli Yang, Shaoqin Zheng, Qin Xu, Jianping Song, Changsheng Deng

**Affiliations:** 1 Institute of Tropical Medicine, Guangzhou University of Chinese Medicine, Guangzhou, 510006 Guangdong People’s Republic of China; 2 Science and Technology Park, Guangzhou University of Chinese Medicine, Guangzhou, 510445 Guangdong People’s Republic of China; 3 School of Stomatology, Jinan University, Guangzhou, 510632 Guangdong People’s Republic of China; 4 Artepharm, Co., Ltd, Guangzhou, 510405 Guangdong People’s Republic of China; 5 Laboratory of Malaria and Vector Research, National Institute of Allergy and Infectious Diseases, National Institutes of Health, Bethesda, MD 20892 USA; 6 National Malaria Control Programme, BP 500, Moroni, Comoros; 7 Ministry of Health Comoros, BP 403, Moroni, Comoros

**Keywords:** Malaria population, Grande Comore, Genetic diversity, Multiplicity of infection, PCR, DNA sequencing

## Abstract

**Background:**

Malaria is still one of the serious public health problems in Grande Comore Island, although the number of annual cases has been greatly reduced in recent years. A better understanding of malaria parasite population diversity and transmission dynamics is critical for assessing the effectiveness of malaria control measures. The objective of this study is to investigate temporal changes in genetic diversity of *Plasmodium falciparum* populations and multiplicity of infection (MOI) in Grande Comore 10 years after introduction of ACT.

**Methods:**

A total of 232 *P. falciparum* clinical isolates were collected from the Grande Comore Island during two sampling periods (118 for 2006‒2007 group, and 114 for 2013‒2016 group). Parasite isolates were characterized for genetic diversity and complexity of infection by genotyping polymorphic regions in merozoite surface protein gene 1 (*msp*-*1*), *msp*-*2*, and *msp*-*3* using nested PCR and DNA sequencing.

**Results:**

Three *msp*-*1* alleles (K1, MAD20, and RO33), two *msp*-*2* alleles (FC27 and 3D7), and two *msp*-*3* alleles (K1 and 3D7) were detected in parasites of both sampling periods. The RO33 allele of *msp*-*1* (84.8%), 3D7 allele of *msp*-*2* (90.8%), and K1 allele of *msp*-*3* (66.7%) were the predominant allelic types in isolates from 2006–2007 group. In contrast, the RO33 allele of *msp*-*1* (63.4%), FC27 allele of *msp*-*2* (91.1%), and 3D7 allele of *msp*-*3* (53.5%) were the most prevalent among isolates from the 2013–2016 group. Compared with the 2006‒2007 group, polyclonal infection rates of *msp*-*1* (from 76.7 to 29.1%, *P* < 0.01) and *msp*-*2* (from 62.4 to 28.3%, *P* < 0.01) allelic types were significantly decreased in those from 2013‒2016 group. Similarly, the MOIs for both *msp*-*1* and *msp*-*2* were higher in *P. falciparum* isolates in the 2006**–**2007 group than those in 2013**–**2016 group (MOI = 3.11 vs 1.63 for *msp*-*1*; MOI = 2.75 vs 1.35 for *msp*-*2*). DNA sequencing analyses also revealed reduced numbers of distinct sequence variants in the three genes from 2006‒2007 to 2013‒2016: *msp*-*1*, from 32 to 23 (about 28% decline); *msp*-*2* from 29 to 21 (about 28% decline), and *msp*-*3* from 11 to 3 (about 72% decline).

**Conclusions:**

The present data showed dramatic reduction in genetic diversity and MOI among Grande Comore *P. falciparum* populations over the course of the study, suggesting a trend of decreasing malaria transmission intensity and genetic diversity in Grande Comore Island. These data provide valuable information for surveillance of *P. falciparum* infection and for assessing the appropriateness of the current malarial control strategies in the endemic area.

## Background

Malaria is a major infectious disease that led to ~ 212 million clinical cases and about 429,000 deaths worldwide in 2016 [1]. *Plasmodium falciparum* malaria had been widely distributed throughout the Union of Comoros (Grande Comore, Moheli, and Anjouan Islands) and posed a serious impediment to socioeconomic development historically [2]. To effectively control malaria in Comoros, many malaria control measures have been deployed since 2000s, including indoor residual sprayings (IRS), long-lasting insecticide nets (LLINs), artemisinin-based combination therapy (ACT), intermittent presumptive treatment (IPT) for all pregnant women, and, particularly, mass drug administration (MDA) of ACT. These malaria control measures have resulted in substantial decrease malaria infection, from 108,260 cases in 2006 to 1072 in 2015 (about 99.0% decline) in Comoros, with no malaria-related deaths. However, despite the great efforts in malaria control, the annual malaria cases increased from 2015 (1072 cases) to 2016 (1372 cases) in Comoros, and the threat of future malaria outbreak remains. Furthermore, malaria transmission intensity differs among the three islands of Comoros (Grande Comore, Moheli, and Anjouan Islands). In Anjouan and Moheli, there was a limited numbers of malaria annual cases during 2014 to 2016 (7 and 5 in 2014; 3 and 8 in 2015; 4 and 6 in 2016, respectively) without local malaria infection; in contrast, the Grande Comore accounted for about 99% of the total of malaria annual cases reported in Comoros during 2013–2016 (e.g. 53,979 in 2013; 2130 in 2014; 1061 in 2015; 1362 in 2016) due to low coverage level of ACT-based MDA. To achieve an ambitious goal of completely eliminating malaria by 2020 in Comoros, there is an urgent need to develop effective and affordable malaria control and treatment strategies.

To date, several malaria pre-erythrocytic (RTS/S and PfSPZ Vaccine) or erythrocytic (MSP-1, MSP-2, and MSP-3) stage vaccines have been designed to induce immunity against the pre-erythrocytic or erythrocytic stage of the malaria parasites, respectively [3, 4]. Although several vaccines are now being tested in clinical Phase I and II trials (MSP-1, MSP-2, and MSP-3) or even have completed the pivotal Phase III clinical testing (RTS/S), the efficacies of these vaccines have been low, with limited impact against clinical malaria [5, 6]. One of the difficulties in developing an effective vaccine against *P. falciparum* parasite is the extensive genetic diversity of vaccine targets allowing parasites with mutated genes to escape from the host’s immune response [7, 8]. Thus, studying genetic diversity of malaria parasites in endemic areas may provide important information to improve vaccine design. Additionally, the genetic diversity of *P. falciparum* parasites has been widely used as an indicator of level of malaria transmission intensity in endemic regions, thus serving as a tool to evaluate the effectiveness of malaria control and intervention.

Polymorphic genetic marks, such as microsatellites and genes encoding merozoite surface proteins (*msp-1*, *msp-2*, and *msp-3*) have been widely used for characterization of parasite genetic diversity [9, 10]. Currently, only one study described the genetic structure of *P. falciparum* parasites collected from Comoros Archipelago (Grande Comore, Moheli, Anjouan, and Mayotte) using microsatellite loci [11], showing that microsatellite genotypes of the *P. falciparum* populations in Grande Comore were substantially different from those in other two islands (Moheli and Anjouan). Currently, no data on temporal changes in genetic diversity of *P. falciparum* isolates from Grande Comoros after introduction of ACT are available. Herein, the objective of this study is to investigate the dynamics of genetic diversity and multiplicity of infection (MOI) in clinical *P. falciparum* isolates from Grande Comore during two different periods (2006‒2007 and 2013‒2016) using polymorphic markers of *msp-1*, *msp-2*, and *msp-3*. The data in this study provide insights on parasite diversity and MOI after various malaria control measures.

## Methods

### Ethics clearance

This study was approved by the Ethics Committees of Comoros Ministry of Health (No. 07-123/VP-MSSPG/DNS) and Guangzhou University of Chinese Medicine (No. 2012L0816). Blood samples were collected from children after obtaining written informed consent from their parents or legal guardians.

### Study sites and sample collection

**Fig. 1:**
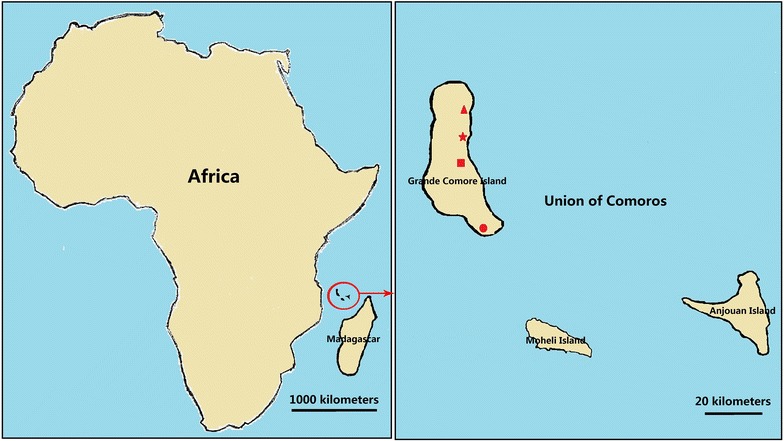
Maps of Grande Comore Island, Union of Comoros, showing the studied locations: Mitsoudje Centre Hospital (triangle), Mbeni District Health Centre (star), National Malaria Centre (square), and Mitsamiouli Centre Hospital (circle)

This study was conducted on the Grande Comore Island, Union of Comoros (Fig. 1), that is located in the Indian Ocean off the south-east coast of Africa, to the east of Mozambique and north-west of Madagascar (11°00′–12°00′S, 43°10′–43°35′E). This island has an area of 1147 km^2^ with about 420,000 inhabitants (2012 estimate). A tropical hot and rainy season occurs from November to April, and a cooler dry season runs from May to October. Annual temperature ranges from 11 to 35 °C and rainfall ranges from 1000 to 3000 mm per year. Malaria transmission on this island is perennial with most of infections occurring during the rainy season. *P. falciparum* is the dominant malaria species, with occasional *Plasmodium malariae* and *Plasmodium vivax* infections [12].

A total of 232 blood samples from microscopically *P. falciparum* positive patients visiting local healthcare facilities were collected in two different periods (March 2006–October 2007 and March 2013–December 2016). Among these samples, 118 samples from Mitsoudje Centre Hospital (50), National Malaria Centre (30), and Mitsamiouli Centre Hospital (38) were collected during March 2006–October 2007; the other 114 samples from Mitsoudje Centre Hospital (41), National Malaria Centre (24), Mitsamiouli Centre Hospital (29), and Mbeni District Health Centre (20) were collected during March 2013–December 2016. Venous blood sample of 1.0 ml was collected from each patient in an EDTA tube and stored at −20 °C until DNA extraction.

### PCR amplification and allelic analysis of the *pfmsp*-*1*, *pfmsp*-*2*, and *pfmsp*-*3* genes

Genomic DNA of each blood sample was extracted using Takara DNA Blood Mini Kit according to the manufacturer’s instructions (Takara, Kyoto, Japan). Extracted parasite DNA was dissolved in TE buffer (10 mM Tris–HCl, 0.1 M EDTA, pH 8.0) and stored in microfuge tubes at −20 °C. Segments of the *pfmsp*-*1* (block 2), *pfmsp*-*2* (block 3), and *pfmsp*-*3* were amplified using nest PCR, as described previously [10, 13]. An initial amplification of the outer regions of the three genes was followed by a nested PCR with sequence specific primer pairs. All the reactions were carried out in final volume of 25 μl containing 10.0 μl of dH_2_O, 0.5 μl of each primer (0.4 µM), 12.5 μl of *Taq* PCR Mast Mix (2.5 U) following the manufacturer’s instructions (Sangon Bio Inc., Shanghai, China) on a S1000 Thermal cycler (Bio-Rad, Hercules, USA). In the primary amplification reactions, 2.0 μl of template genomic DNA were added as a temple. In the nested reaction, 0.5 μl of primary PCR product was added as a temple. The nested PCR products were separated on 2.0% agarose gel (Sangon Bio Inc., Shanghai, China) and visualized under ultraviolet (UV) trans-illumination. A 100-bp DNA ladder was used to determine the size of PCR products (Sangon Bio Inc., Shanghai, China). MOI of the *msp*-*1* or *msp*-*2* genes was calculated by averaging number of amplified bands per positive *P. falciparum* isolate as described previously [14]. For DNA sequencing, amplified DNA fragments representing different alleles were purified using a PCR purification kit (Takara, Kyoto, Japan). Purified PCR products from selected isolates representing different alleles of *msp*-*1*, *msp*-*2*, and *msp*-*3* were directly sequenced in both directions with the primers in the secondary PCR using an ABI PRISM3730 DNA sequencer (Sangon Bio Inc., Shanghai, China). The sequences were also used to correct the estimated molecular weight and to confirm the nature of the amplified product.

### Statistical analysis

Statistical analysis was determined using SPSS (version 13.0) software. Comparisons of MOI of *msp*-*1* or *msp*-*2* in isolates collected between 2006–2007 and 2013–2016 were made using *t* test. Mann–Whitney U test was used to compare in the frequencies of the mutations and alleles of the *msp*-*1*, *msp-2* and *msp-3* in isolates collected between 2006–2007 and 2013–2016. *P* < 0.05 was considered indicative of a statistically significant difference.

## Results

### Allelic polymorphism of *msp*-*1* and msp*-2*

Approximately 98% of *P. falciparum* isolates (222/232) were successfully amplified from *msp*-*1* block 2 region (112 for 2006–2007 group; and 110 for 2013–2016 group), and three allelic types (K1, MAD20, and RO33) were identified from the samples (Table 1). The band sizes were 150–300 bp for the K1 type, 140–280 bp for the MAD20 type, and only one band size (~ 150 bp) for RO33 allelic type. For 2006–2007 group, 58 isolates (51.8%) were K1 type; 48 (42.9%) were MAD20 type; and 95 (84.8%) were RO33 type. For the 2013–2016 group, 46 (41.8%), 26 (23.6%), and 70 (63.4%) isolates had K1, MAD20, and RO33 allelic types, respectively. For individual infections among the 2006–2007 group, 6.3% carried K1 type, 2.7% carried MAD20 type, 14.3% carried RO33 type, and 76.7% had multiple allelic types (i.e. K1/MAD20, K1/RO33, MAD20/RO33, and K1/MAD20/RO33). In contrast, 23.6% of the 2013–2016 infections had K1 type, 12.7% had MAD20 type, 34.5% had RO33 type, and 29.1% contained multiple allelic types (i.e. K1/MAD20, K1/RO33, and MAD20/RO33). Compared with the 2006–2007 group, the frequencies of isolates with multiple allelic types was significantly decreased (from 76.7 to 29.1%, *P* < 0.01) in those from 2013–2016 group, whereas the frequencies of isolates with only K1, MAD20, or RO33 allelic types was significantly increased (*P* < 0.01).

**Table 1:** Prevalence and multiplicity of infection (MOI) of *msp*-*1* and *msp*-*2* allelic types in *Plasmodium falciparum* isolates from Grande Comore Island in two different periods

Gene types	Isolates collected in 2006–2007 period		Isolates collected in 2013–2016 period	
	No. of samples (%)	MOI	No. of samples (%)^a^	MOI^b^
*msp*-*1*				
K1	7 (6.3)	2.28	26 (23.6)**	1.70^##^
MAD20	3 (2.7)	2.33	14 (12.7)**	1.79^##^
RO33	16 (14.3)	2.50	38 (34.5)**	1.34^##^
K1 + MAD20	7 (6.3)	2.29	0 (0)**	0^##^
K1 + RO33	41 (36.7)	3.46	20 (18.2)**	1.70^##^
MAD20 + RO33	35 (31.3)	3.43	12 (10.9)**	2.08^##^
K1 + MAD20 + RO33	3 (2.7)	2.67	0 (0)	0^##^
Total	112 (100)	3.11	110 (100)	1.63^##^
*msp*-*2*				
FC27	10 (9.2)	2.30	71 (62.8)**	1.20^##^
3D7	31 (28.4)	2.90	10 (8.8)**	2.30^#^
FC27 + 3D7	68 (62.4)	2.75	32 (28.3)**	1.41^##^
Total	109 (100)	2.75	113 (100)	1.35^##^

^**a**^Statistically significant differences for comparison with isolates circulating in 2006–2007 from Grande Comore island (* *P* < 0.05; ** *P* < 0.01) using Mann–Whitney *U* test

^**b**^Statistically significant differences for comparison with isolates circulating in 2006–2007 from Grande Comore island (^#^ *P* < 0.05; ^##^ *P* < 0.01) using t test

A total of 222 *P. falciparum* isolates had a positive PCR outcome for *msp*-*2* block 3, including 109 samples collected during 2006–2007 and 113 samples during 2013–2016. Two allelic types (FC27 and 3D7) were identified in both two periods (Table 1), with PCR product sizes varying from 250 to 550 bp for FC27 allelic type and from 400 to 660 bp for 3D7 allelic type, respectively. For samples collected during 2006–2007, 71.6 and 90.8% carried FC27 and 3D7 allelic types, while 91.1 and 37.1% of the 2013–2016 samples had FC27 and 3D7 allelic types, respectively. For individual infections, 9.2% contained only FC27 type, 28.4% carried only 3D7 type, and the remaining 62.4% had two allelic types in 2006–2007 group (i.e. FC27/3D7). For 2013–2016 group, 62.8% of the infections contained only FC27 type, 8.8% carried only 3D7 type, and the remaining 28.3% had multiple allelic types (i.e. FC27/3D7), again showing a decreasing trend in mixed genotype infection (62.4% in 2006–2007, and 28.3% in 2013–2016; *P* < 0.01). MOIs for both *msp*-*1* (3.11) and *msp*-*2* (2.75) were higher in *P. falciparum* isolates in 2006**–**2007 group than those in 2013**–**2016 group (MOI = 1.63 and 1.35, respectively).

### Sequence analysis of the *msp*-*1* gene

**Fig. 2:**
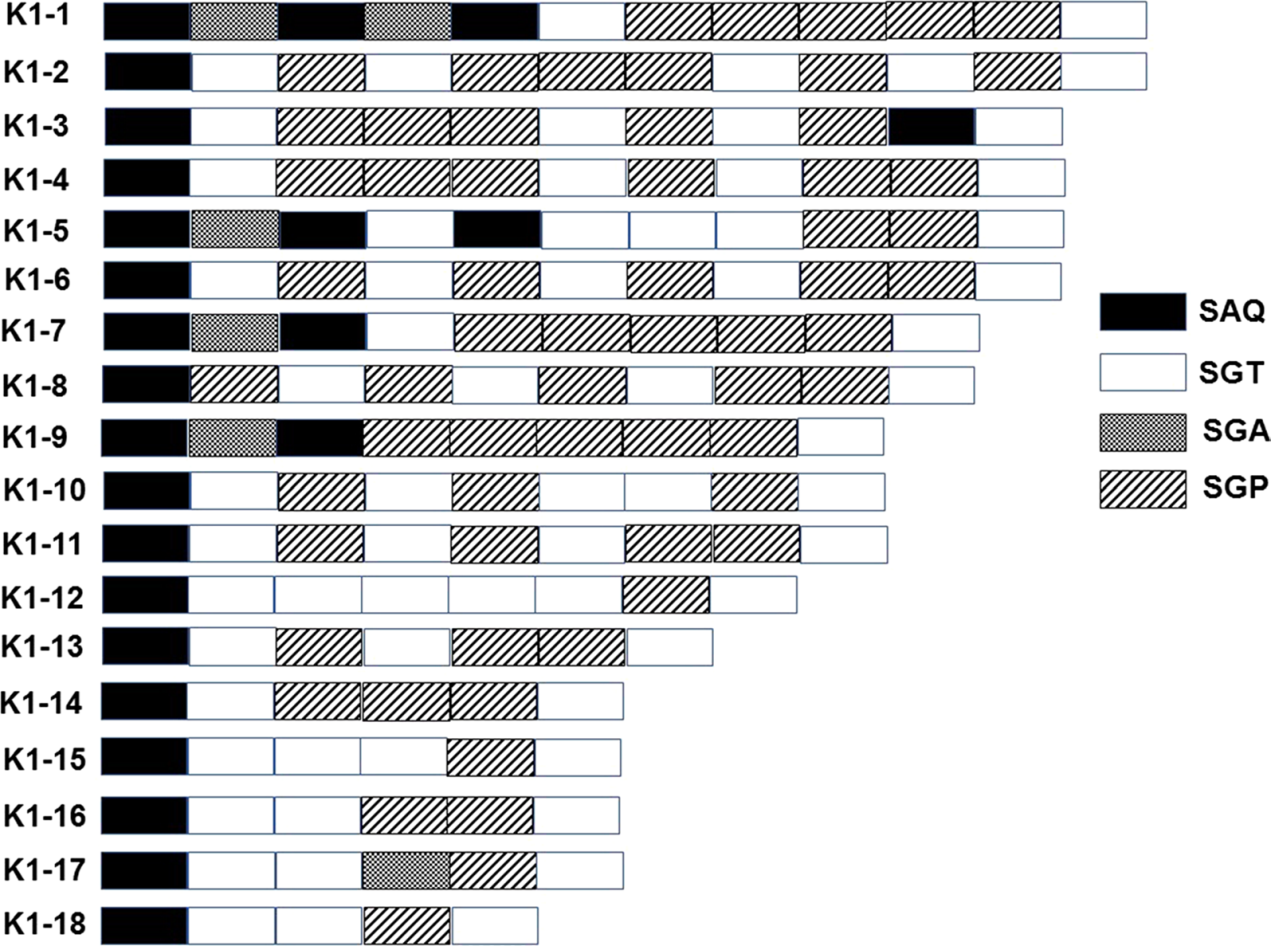
Schematic representation of *msp*-*1* K1 distinct allelic variants of Grande Comore *Plasmodium falciparum* isolates. There were 18 distinct variants based on the number and arrangement of SAQ, SGT, SGA and SGP motifs, with 14 from the 2006‒2007 group (K1-1, 3, 4, 6, 7, 9, 10–13, and 15–18) and 11 from the 2013‒2016 group (K1-2, 5–9, 11–14, and 18)

**Fig. 3:**
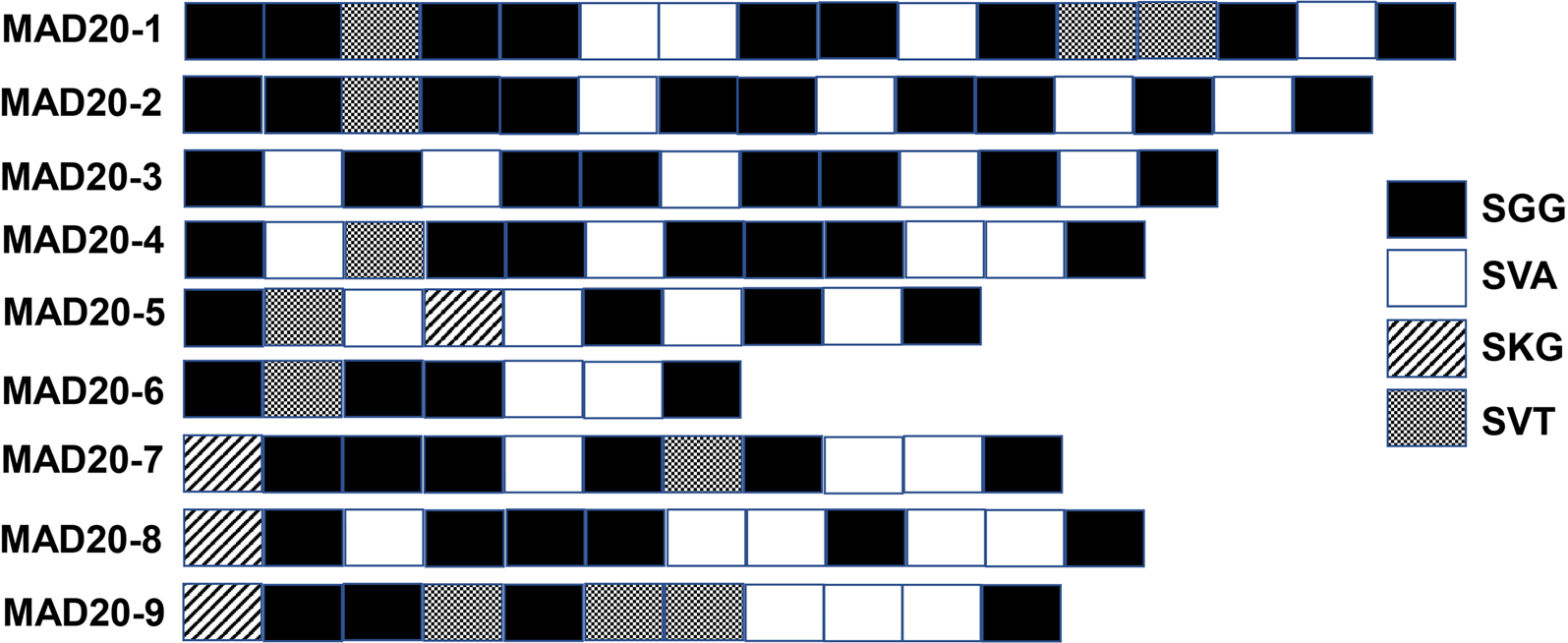
Schematic representation of *msp*-*1* MAD20 allelic types of Grande Comore *Plasmodium falciparum* isolates. A total of 9 distinct variants were detected based on the number and arrangement of SGG, SVT, SKG, and SVA motifs, with 9 from the 2006‒2007 group (MAD20-1 to 9) and 3 from the 2013‒2016 group (MAD20-1 to 3)

**Fig. 4:**
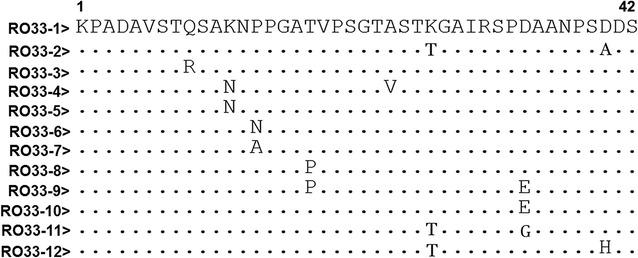
Sequence alignments of amino acid sequences of *msp*-*1* RO33 distinct allelic variants among *Plasmodium falciparum* isolates from Grande Comore. A total of 12 distinct variants were found by sequence analysis of *msp*-*1* block 2, including 9 from the 2006‒2007 group (RO33-1 to 6, 8, 10, and 12) and 9 form the 2013‒2016 group (RO33-1 to 5, 7, 9, 11, and 12). Dots and dashes represent identical residues and deletions, respectively

Sequence analysis of *msp*-*1* block 2 region revealed that the Grande Comore isolates contained a limited number of different tripeptide repeat units (SAG, SGT, SGA, and SGP for K1 allelic type; and SGG, SVA, SKG, and SVT for MAD20 allelic type). For the K1 allelic type, the tripeptide repeat region always started with SAQ and terminated with SGT (Fig. 2); while the tripeptide repeat region of the MAD20 type alleles usually started with one of two tripeptide repeat units (SGG or SKG) and always ended with two tripeptide repeat units (SVASGG) (Fig. 3). The allelic diversity in K1 and MAD20 allelic types could be mainly caused by duplications or deletions of these repeat motifs. For the RO33 allelic type, the polymorphisms could be mainly due to amino acid substitutions in the KDGANTQVVAKPA/DAVSTQSAKNPPGATVPSGTASTKGAIRSPGAANPS sequences (Fig. 4).

Sequence analysis of *msp*-*1* block 2 region showed that the isolates could be divided into three allelic types (K1, MAD20, and RO33) (Table 2). In total, 32 distinct sequence variants were found in 2006–2007 group, with 14 variant subtypes for the K1 type, 9 variant subtypes for MAD20, and 9 variant subtypes for RO33. Of these variant subtypes, K1-18 (20.0%), MAD20-1 (25.0%), and RO33-3 (24.2%) subtypes were the most prevalent in the 2006–2007 group. For the 2013–2016 group, a total of 23 distinct variants in *msp*-*1* gene were detected: 11 variant subtypes for the K1 type, 3 for MAD20 type, and 9 for RO33 type. Of the 23 sequence variants, the K1-13, MAD20-1/MAD20-3, and RO33-1 subtypes were the most prevalent, accounting for 20.5, 41.6, and 59.3%, respectively. Compared with the 2006–2007 group, the frequencies of *msp*-*1* K1-1 (*P* < 0.05), K1-4 (*P* < 0.01), K1-6 (*P* < 0.01), K1-10 (*P* < 0.01), K1-15 (*P* < 0.05), K1-17 (*P* < 0.01), and K1-18 (*P* < 0.05) subtypes were significantly decreased in the 2013–2017 group, whereas the K1-2 (*P* < 0.05), K1-5 (*P* < 0.01), K1-8 (*P* < 0.01), K1-11 (*P* < 0.01), K1-13 (*P* < 0.01), and K1-14 (*P* < 0.05) were significantly increased. For *msp*-*1* MAD20 allelic types, significant decrease in MAD20-4 to MAD20-9 allelic types (*P* < 0.05 or *P* < 0.01) and significant increase in MAD20-1 to MAD20-3 types (*P* < 0.01) were observed in 2013–2016 group compared with those of the 2006–2007 group. Similarly, in comparison to 2006–2007 group, significantly reduced frequencies of RO33-3, RO33-5, RO33-6, RO33-8, and RO33-10 were observed in the 2013–2016 group (*P* < 0.01), whereas significantly increased of RO33-1 (*P* < 0.01), RO33-7 (*P* < 0.05), and RO33-11 (*P* < 0.01) types were detected in the 2013–2016 group.

**Table 2:** Prevalence of *msp*-*1* K1, MAD20, and RO33 allelic variants collected from *Plasmodium falciparum* isolates along Grande Comore Island in 2006–2007 and 2013–2016 periods

K1 allele	Number of isolates (%)^a^		MAD20 allele	Number of isolates (%)^a^		RO33 allele	Number of isolates (%)^a^	
	2006–2007	2013–2016		2006–2007	2013–2016		2006–2007	2013–2016
	(n = 40)	(n = 44)		(n = 20)	(n = 12)		(n = 42)	(n = 54)
K1-1	2 (5.0)	0 (0)*	MAD20-1	5 (25.0)	5 (41.6)**	RO33-1	7 (21.2)	32 (59.3)**
K1-2	0 (0)	2 (4.5)*	MAD20-2	1 (5.0)	2 (16.6)**	RO33-2	2 (6.1)	3 (5.6)
K1-3	1 (2.5)	0 (0)	MAD20-3	2 (10.0)	5 (41.6)**	RO33-3	8 (24.2)	1 (1.9)**
K1-4	3 (7.5)	0 (0)**	MAD20-4	3 (15.0)	0 (0)**	RO33-4	2 (6.1)	1 (1.9)
K1-5	0 (0)	5 (11.4)**	MAD20-5	2 (10.0)	0 (0)**	RO33-5	4 (12.1)	1 (1.9)**
K1-6	3 (7.5)	2 (4.5)	MAD20-6	2 (10.0)	0 (0)**	RO33-6	2 (6.1)	0 (0)**
K1-7	5 (12.5)	5 (11.5)	MAD20-7	1 (5.0)	0 (0)*	RO33-7	0 (0)	3 (5.6)*
K1-8	0 (0)	3 (6.8)**	MAD20-8	1 (5.0)	0 (0)*	RO33-8	3 (9.1)	0 (0)**
K1-9	2 (5.0)	2 (4.5)	MAD20-9	3 (15.0)	0 (0)**	RO33-9	0 (0)	2 (3.7)
K1-10	3 (7.5)	0 (0)**				RO33-10	3 (9.1)	0 (0)**
K1-11	2 (5.0)	8 (18.2)**				RO33-11	0 (0)	8 (14.8)**
K1-12	2 (5.0)	2 (4.5)				RO33-12	2 (6.1)	3 (5.6)
K1-13	3 (7.5)	9 (20.5)**						
K1-14	0 (0)	2 (4.5)*						
K1-15	2 (5.0)	0 (0)*						
K1-16	1 (2.5)	0 (0)						
K1-17	3 (7.5)	0 (0)**						
K1-18	8 (20.0)	4 (9.1)*						

^**a**^Statistically significant differences for comparison with isolates circulating in 2006–2007 from Grande Comore island (* *P* < 0.05; ** *P* < 0.01) using Mann–Whitney *U* test

### Sequence analysis of the *msp*-*2* gene

**Fig. 5:**
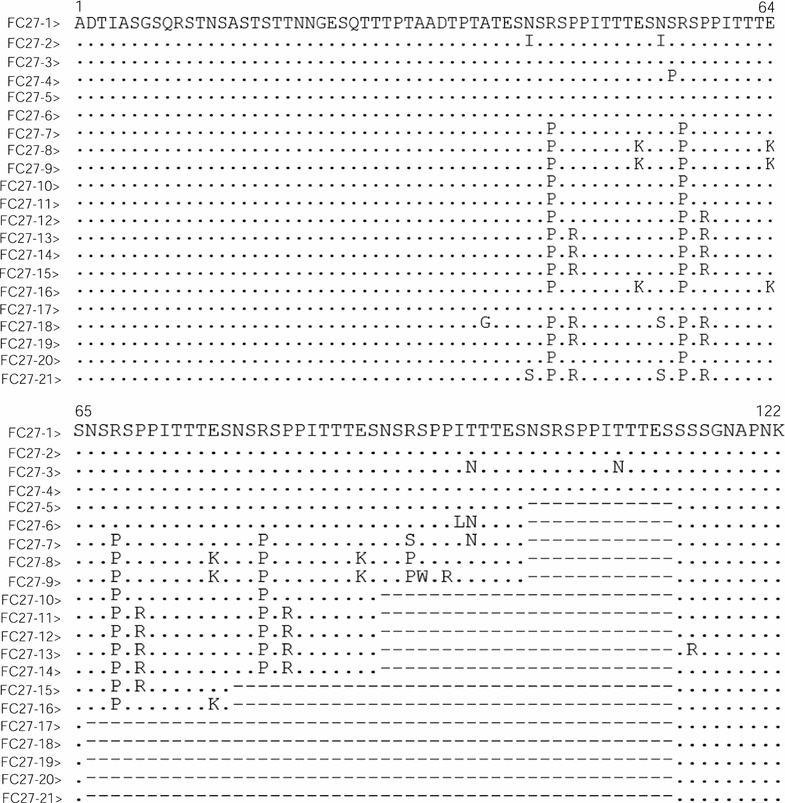
Sequence alignments of amino acid sequences of *msp*-*2* FC27 distinct allelic variants of *Plasmodium falciparum* isolates in Grande Comore. A total of 21 distinct variants were identified by sequence analysis of *msp*-*2* block 3 in two groups, including 16 for the 2006‒2007 group (FC27-1 to 3, 5–7, 9–14, 16, and 19–21) and 13 for the 2013‒2016 group (FC27-1 to 4, 8–10, 12–15, 17, and 18). Dots and dashes represent identical residues and deletions, respectively

**Fig. 6:**
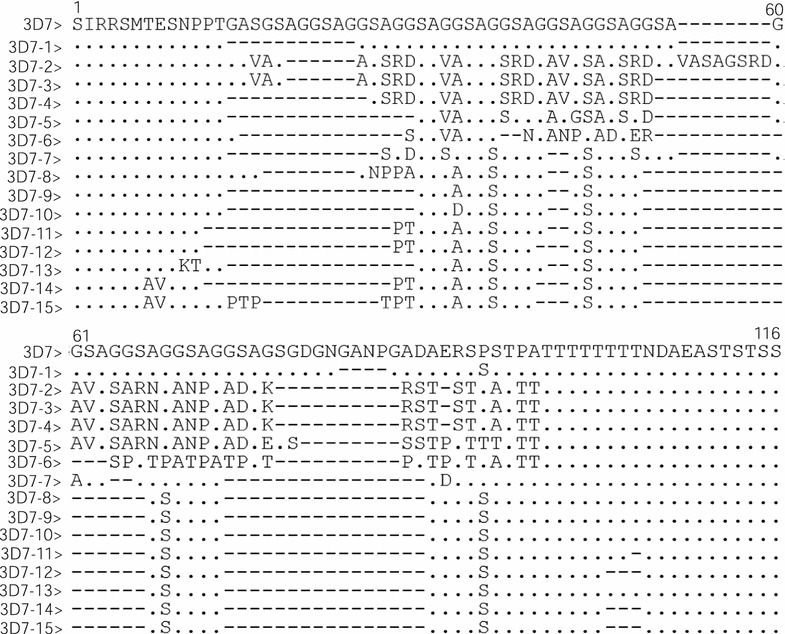
Sequence alignments of amino acid sequences of *msp*-*2* 3D7 distinct allelic variants of *Plasmodium falciparum* isolates in Grande Comore. A total of 15 distinct variants were identified by sequence analysis of *msp*-*2* block 3, including 13 for the 2006‒2007 group (3D7-1 to 6, 8–11, and 13–15) and 8 for the 2013‒2016 group (3D7-1 to 3, 7–9, 11, and 12). Dots and dashes represent identical residues and deletions, respectively

Sequence analysis of *msp*-*2* block 3 region showed that the FC27 alleles contained a family-specific region (ADTIASGSQSSTNSASTSTTNNGESQTTTPTA), a conserved 8 amino acid region (ADTPTATE), a 12 amino acid repeating unit (SNSRSPPITTE or SNSPSPPITITE, n = 0–4), and followed by a conserved region (SSGNAPNK) at the end (Fig. 5). The polymorphisms of FC27 allelic type were mainly due to the non-synonymous amino acid changes in these regions, whereas the polymorphic character of 3D7 allelic type were mainly variable repeating units of 4–10 amino acids as well as different numbers (n = 5–8) of threonine residues (T) (Fig. 6).

Fragments of the *P. falciparum msp*-*2* block 3 were successfully amplified and sequenced from 118 samples of the 2006–2007 group and 111 samples of the 2013–2016 group. Amino acid sequence analysis revealed that 52.5% (62/118) and 47.5% (56/118) of the samples had FC27 and 3D7 allelic types in the 2006–2007 group (Table 3), with a total of 26 distinct haplotypes (16 for FC27 allelic type; and 13 for 3D7 allelic type). For the 2006–2007 group, the FC27-5/10 (12.9%) and 3D7-2 (34.0%) haplotype was the most abundant. However, in the 2013–2016 group, 80 (72.1%) and 33 (27.9%) samples had the FC27 and 3D7 allelic types of *msp*-*2*, respectively. There were 13 different FC27 haplotypes and 8 3D7 haplotypes in the 2013–2016 group (Table 3). Of these haplotypes, the FC27-14 and 3D7-9 were the most abundant, with an overall frequency of 58.8 and 48.5%, respectively. Compared with the 2006–2007 group, the frequencies of FC27-5 (*P* < 0.01), FC27-6 (*P* < 0.01), FC27-7 (*P* < 0.01), FC27-10 (*P* < 0.01), FC27-11 (*P* < 0.01), FC27-16 (*P* < 0.01), and FC27-19 (*P* < 0.01), and 3D7-2 (*P* < 0.01), 3D7-4 (*P* < 0.01), 3D7-5 (*P* < 0.05), 3D7-13 (*P* < 0.01), and 3D7-15 (*P* < 0.05) were significantly decreased in the 2013–2016 group, whereas the frequencies of FC27-14, 3D7-7, 3D7-9, and 3D7-12 haplotypes were significantly increased (*P* < 0.01). Some of the *P. falciparum* Grande Comore isolates with *msp*-*2* FC27 and 3D7 haplotypes in this study showed 100% identity with other strains from Vietnam (AAG47596 with FC27-20), while other haplotypes (FC27-1 to FC27-19, FC27-19, 3D7-1 to 3D7-15) were new alleles identified in this study.

**Table 3:** Prevalence of *msp*-*2* FC27 and 3D7 allelic variants collected from *Plasmodium falciparum* isolates along Grande Comore Island in 2006–2007 and 2013–2016 periods

FC27 allele	Number of isolates (%)^a^		3D7 allele	Number of isolates (%)^a^	
	2006–2007 (n = 62)	2013–2016 (n = 80)		2006–2007 (n = 56)	2013–2016 (n = 33)
FC27-1	4 (6.4)	8 (10.0)	3D7-1	4 (7.1)	2 (6.1)
FC27-2	1 (1.6)	3 (3.8)	3D7-2	19 (34.0)	2 (6.1)**
FC27-3	2 (3.2)	2 (2.5)	3D7-3	3 (5.4)	3 (9.1)
FC27-4	0 (0)	3 (3.7)	3D7-4	4 (7.2)	0 (0)**
FC27-5	8 (12.9)	0 (0)**	3D7-5	3 (5.4)	0 (0)*
FC27-6	3 (4.8)	0 (0)**	3D7-6	1 (1.8)	0 (0)
FC27-7	6 (9.7)	0 (0)**	3D7-7	0 (0)	3 (9.1)**
FC27-8	0 (0)	2 (2.5)	3D7-8	3 (5.4)	2 (6.1)
FC27-9	2 (3.2)	3 (3.8)	3D7-9	6 (10.7)	16 (48.5)**
FC27-10	8 (12.9)	2 (2.5)**	3D7-10	2 (3.6)	0 (0)
FC27-11	4 (6.5)	0 (0)**	3D7-11	3 (5.4)	2 (6.1)
FC27-12	3 (4.8)	2 (2.5)	3D7-12	0 (0)	3 (9.1)**
FC27-13	3 (4.8)	3 (3.8)	3D7-13	4 (7.1)	0 (0)**
FC27-14	7 (11.3)	47 (58.8)**	3D7-14	1 (1.8)	0 (0)
FC27-15	0 (0)	2 (2.5)	3D7-15	3 (5.4)	0 (0)*
FC27-16	5 (8.1)	0 (0)**			
FC27-17	0 (0)	1 (1.3)			
FC27-18	0 (0)	2 (2.5)			
FC27-19	3 (4.8)	0 (0)**			
FC27-20	1 (1.6)	0 (0)			
FC27-21	2 (3.2)	0 (0)			

^**a**^ Statistically significant differences for comparison with isolates circulating in 2006–2007 from Grande Comore island (* *P* < 0.05; ** *P* < 0.01) using Mann–Whitney *U* test

### Sequence analysis of *msp*-*3*

**Fig. 7:**
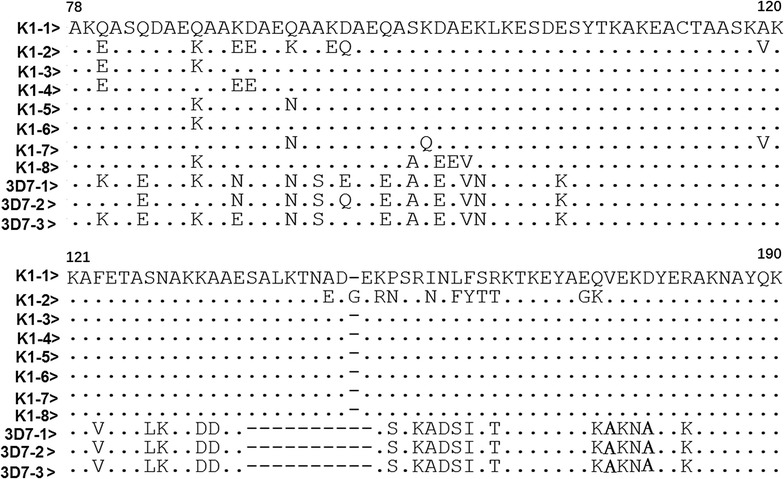
Sequence alignments of amino acid sequences of *msp*-*3* distinct allelic variants of *Plasmodium falciparum* isolates in Grande Comore. A total of 11 distinct variants were found, including 11 for the 2006‒2007 group (K1-1 to 8; and 3D7-1 to 3) and 3 for the 2013‒2016 group (K1-1 and 2; and 3D7-1). Dots and dashes represent identical residues and deletions, respectively

Of the 232 confirmed *P. falciparum* samples obtained from Grande Comore, 204 were successfully amplified and sequenced for *msp*-*3* (105 for the 2006–2007 group and 99 for the 2013–2016 group). Sequence analysis of *msp*-*3* showed that the allelic diversity of *msp*-*3* allelic types (3D7 and K1) was attributed to variation in size of the AHR region, with K1 allelic type being 366 or 369 bp long and 3D7 type having 339 bp (Fig. 7). For the 2006–2007 group, 66.7% (70/105) and 33.3% (35/105) of the samples contained K1 and 3D7 allelic types, with 8 (K1-1 to K1-8) and 3 (3D7-1 to-3D7-3) distinct haplotypes, respectively (Table 4). Of these haplotypes, the K1-5 and 3D7-1 were the most abundant haplotypes, with an overall frequency of 20.0 and 15.2%, respectively. In contrast, 46 (46.5%) and 53 (53.5%) samples in the 2013–2016 group had K1 and 3D7 allelic types, respectively (Table 4). There were only two different K1 haplotypes (K1 and K2) and one 3D7 haplotype in the 2013–2016 group. The K1-2 and 3D7-1 were the most abundant haplotypes, accounting for 37.4.0 and 53.5% of the isolates examined, respectively. Compared with the 2006–2007 group, the frequencies of K1-3, K1-5, K1-6, 3D7-2 and 3D7-3 was significantly decreased in 2013–2014 group (*P* < 0.01), whereas the frequencies of K1-2 and 3D7-1 haplotypes was significantly increased (*P* < 0.01).

**Table 4:** Prevalence of *msp*-*3* K1 and 3D7 allelic variants collected from *Plasmodium falciparum* isolates along Grande Comore Island in 2006–2007 and 2013–2016 periods

Allelic variants	Number of isolates (%)^a^		
	2006–2007 (n = 105)	2013–2016 (n = 99)	Total (n = 204)
K1-1	4 (7.6)	9 (9.1)	13 (6.4)
K1-2	12 (11.4)	37 (37.4)**	49 (24.0)
K1-3	12 (11.4)	0 (0)**	12 (5.9)
K1-4	2 (1.9)	0 (0)	2 (1.0)
K1-5	16 (15.2)	0 (0)**	21 (10.3)
K1-6	12 (11.4)	0 (0)**	12 (5.9)
K1-7	3 (2.9)	0 (0)	3 (1.5)
K1-8	4 (3.8)	0 (0)	4 (2.0)
3D7-1	21 (20.0)	53 (53.5)**	69 (33.8)
3D7-2	12 (10.4)	0 (0)**	12 (5.9)
3D7-3	7 (3.8)	0 (0)**	7 (3.4)

^**a**^Statistically significant differences for comparison with isolates circulating in 2006–2007 from Grande Comore island (* *P* < 0.05; ** *P* < 0.01) using Mann–Whitney *U* test

## Discussion

Dramatic reduction in annual malaria cases has been achieved in Grande Comore through the use of ACT for the treatment of uncomplicated *P. falciparum* patients, ACT-based MDA, and other malaria control interventions. However, malaria continues to be one of most important public health problems on this island, which calls for monitoring changes in drug resistance status and parasite population dynamics. Determining *P. falciparum* genetic diversity and MOI from field samples is important for understanding the impacts of malaria control measures on parasite populations and for developing strategies to better control malaria infection. The present study investigates the temporal change of genetic diversity and MOI of Grande Comore *P. falciparum* populations based on *msp*-*1*, *msp*-*2*, and *msp*-*3* genes that have been used to monitor parasite population widely [10, 15]. The present data showed that the frequencies of allelic diversity and MOI of *msp*-*1*, *msp*-*2*, and *msp*-*3* significantly decreased in the 2013–2016 group when compared with those the 2006–2007 group, which may reflect a trend associated with decreasing malaria transmission intensity on this island.

Some *msp*-*1* K1, MAD20, or RO33 haplotypes found in the present study have been reported in other regions of the world, including Brazil (AFS44739 with K1-5), Senegal (ABS84524 with K1-5, ABS84428 with K1-6, and ABS84432 with K1-11), Malawi (ADQ74224 with K1-12 and ADQ74227 with RO33-1), India (AFR61077 with K1-12 and AFR61093 with MAD20-3), Tanzania (BAM84405 with K1-12 and AAC69748 with RO33-1), Colombia (ACS26173 with MAD20-6), and Myanmar (ACB69813 with MAD20-6); whereas the other allele variants of *msp*-*1* were new alleles identified in this study. The present data reveal RO33 being the dominant allelic type of *msp-1* gene among Grande Comore *P. falciparum* isolates in both groups. RO33 has also been reported to the dominant allele in parasites collected from Malaysia [13], Brazil [16], and Gabon [17], whereas MAD20 allele was the most prevalent in Myanmar [18, 19], Thailand [18], Iran [20], Pakistan [21], and Colombia [22], Senegal [23, 24]. For parasites from French Guiana [25], Kenya [26], and Peru [27], K1 was the dominant *msp*-*1* allelic type. For *msp*-*2*, the 3D7 type was the predominant among isolates in 2006–2007 group, similar to reports from several African countries, including Gambia [28], Cameroon [29], Congo [30], Ghana [31, 32], Senegal [14, 23, 24], Burkina Faso [33], Malawi [33], Uganda [33], and Tanzania [34]. The 3D7 allele was also major type in some Southeast Asian countries such as Cambodia [35], Iran [20], Malaysia [13, 36], Myanmar [19], Pakistan [21], Papua New Guinea [37], Thailand [18], as well as Thai-Myanmar borders [38]. In contrast, the FC27 was the dominant allele for parasites from Gabon [39], Cameroon [40], Nigeria [41]. Several previous reports indicate that the FC27 allele is associated with disease severity [42], and the 3D7 type may be the common genotype circulating in high disease transmission areas [32]. Here the data in this study show that over 10 years after the introduction of ACT, the prevalence of 3D7 allelic type in *msp*-*2* gene is dramatically decreased, from 90.8 to 37.1%, whereas FC27 allelic type increased from 71.6 to 91.1%.

Previous reports show that polyclonal infection is more common in areas with high endemicity, and 50–100% of infections are polyclonal infections in mesoendemic and holoendemic areas [43–45]. Furthermore, a significant association between the complexity of infection and polyclonal infections with the asymptomatic malaria was observed in malaria endemic area of Congo [46]. In the present study, more than 76 and 62% of the samples examined harboured polyclonal infections (two or three allelic types) of the *msp*-*1* and *msp*-*2* gene, respectively, in 2006–2007 group. The frequencies of polyclonal infections were reduced to about 29 and 28%, respectively, in the 2013–2016 group, which again suggests decreasing population diversity and/or transmission intensity. MOI is conventional index to measure of complexity of infection and intensity of transmission. A high MOI value is often observed in a hyperendemic region with high malaria transmission [21, 31, 47, 48]. In the present study, the MOI values decreased from 3.11 to 1.63 for *msp*-*1* and from 2.75 to 1.35 for *msp*-*2*, respectively. The findings in this study are similar to those reported in southeastern Senegal [24] and Congo [30]. The present data suggest a progressive decrease of *P. falciparum* transmission on this island. In fact, according to a report from the Comoros Ministry of Health, the numbers of annual malaria cases in Grande Comore dramatically decreased from 92,480 (in 2006) with high level incidence (about 35%) to 1362 (in 2016) with low level incidence (about 0.3%). Therefore, the present data confirm that MOI can be used as a useful indicator for monitoring malaria transmission level in the endemic areas.

Sequence analysis revealed that the decline in the number of *msp*-*1* haplotypes (32 for the 2013–2016 and 23 for the 2006–2007 group) among Grande Comore isolates. Similarly, the total number of haplotypes in *msp*-*2* dramatically decreased from 29 in the 2006–2007 group (16 for FC27 and 13 for 3D7 allelic types) to 21 (13 for FC27 and 8 for 3D7 allelic types) in 2013–2016 group (about 28% decline). This is in agreement with previous reports in other countries with declining endemicity [49, 50]. However, studies from Senegal, Mozambique, and Iran indicated that the introduction of ACT in Congo has reduced the MOI but not the genetic diversity of *msp*-*2* gene among *P. falciparum* isolates from children living in Southern districts of Brazzaville [30]. Again, haplotype analysis supports reduced genetic diversity and transmission on the Grande Comore island.

The polymorphism of *pfmsp*-*3* is predominantly confined to sequence diversity in the N-terminal domain within the heptad-repeats (insertion/deletion and nucleotide substitutions) [10]. The present study detected both *msp*-*3* K1 and 3D7, but not recombinant type, similar to those reported from Thailand, Papua New Guinea, India, Keyan [10]. Recombinant *msp*-*3* alleles were detected in Iran and African countries at a very low frequency [51, 52]. Some of the Grande Comore parasites collected in this study had new msp-3 alleles (K1-3 to K1-8, 3D7-2 and 3D7-3) that have not been reported previously. However, many parasites showed 100% identity with those from Asia, Africa, and South America reported previously, such as Thailand (AOT86948 with K1-1, AOT86951 with K1-2, and AOT86944 with 3D7-1), India (AEI28718 with K1-1, AEI28725 with K1-2, and AEI28765 with 3D7-1), Kenya (AMM75906 with K1-1, AMM75893 with K1-2, and AMM75927 with 3D7-1), Nigeria (CAJ44166 with K1-1, CAJ44194 with K1-2, and CAJ44184 with 3D7-1), China (AAF04099 with K1-1), Indonesia (AAF59914 with K1-2), Papua New Guinea (AAC47670 with K1-2, and AAC47662 with 3D7-1), Vietnam (AAK94780 with K1-2), and Brazil (AFP75269 with K1-2). In the present study, the *msp*-*3* 3D7-1 haplotype was the most prevalent in both 2006–2007 and 2013–2016 groups. The present data were in line with the findings from Thailand, India, and Nigeria, where 3D7-1 haplotypes was the most abundant types [10]. In the present study, K1 allelic type was the predominant (66.7%) in 2006–2007 group. The data in the present study are in some degree consistent with the reports of K1 being the most prevalent type in the Thailand [10], Thailand–Myanmar border [53], and Thailand–Cambodia border [53], but is contrast to previous reports from in Thailand–Laos border [53] and Peru [54], with the 3D7 being the most prevalent type. Over the course of 10 years (from 2006 to 2016), the frequencies of K1 type dramatically decreased from 66.7 to 46.5% (*P* < 0.01), while the 3D7 type dramatically increased from 33.3 to 53.5% (*P* < 0.01), suggesting that parasites with the *msp*-*3* 3D7 type may survive better after introduction of ACTs in Grande Comore. The total number of haplotypes in *msp*-*3* gene changed from 11 in 2006–2007 to 3 in 2013–2016 (a 60% decline), suggesting a decreasing tend in genetic diversity of *msp*-*3* in Grande Comore after 10 years of use of ACT.

The observation of increased frequencies of *msp-2* FC27 and *msp-3* 3D7 allelic types when general population genetic diversity and other allelic types have decreased are interesting, although we do not know the reason for the shift of the alleles. One remote possibility is that the *msp*-*2* FC27 and/or *msp*-*3* 3D7 alleles or some unknown genes nearby (linked to *msp*-*2* and/or *msp*-*3*) play a role in parasite response to ACT. Parasites carrying these specific alleles/genes can survive better under drug pressure and increase frequency. Another possibility is that the 2013–2016 parasite populations might consist of some parasites carrying these alleles imported from nearby endemic regions after reduction in the original parasite populations. These issues require further investigations.

## Conclusion

This study investigated the temporal change in genetic diversity and MOI of *P. falciparum* populations in Grande Comore Island after the introduction of ACT using the polymorphic genetic markers (MSP-1, MSP-2, and MSP-3). Results from the current study showed that the prevalence of genetic diversity and MOI in *msp*-*1*, *msp*-*2*, or *msp*-*3* decreased over the course of the study (July 2006 to July 2016). The data in this study suggest a progressive decrease in genetic diversity likely due to lower malaria transmission intensity. The data presented here provide a valuable information for assessing the appropriateness of the current malarial control strategies in this endemic area.

## Abbreviations

MOI: multiplicity of infection
*msp*-*1*: *P. falciparum* merozoite surface protein gene 1
*msp*-*2*: *P. falciparum* merozoite surface protein gene 2
*msp*-*3*: *P. falciparum* merozoite surface protein gene 3
ACT: artemisinin-based combination therapy

## References

[1] WHO (2016). World malaria report 2016.

[2] Ouledi A (1995). Epidemiology and control of malaria in the Federal Islamic Republic of Comoros (in French). Sante.

[3] Hoffman S, Vekemans J, Richie T, Duffy P (2015). The march toward malaria vaccines. Vaccine.

[4] Matuschewski K (2017). Vaccines against malaria-still a long way to go. FEBS J.

[5] Gosling R, von Seidlein L (2016). The future of the RTS, S/AS01 malaria vaccine: an alternative development plan. PLoS Med.

[6] Beeson J, Drew D, Boyle M, Feng G, Fowkes F, Richards J (2016). Merozoite surface proteins in red blood cell invasion, immunity and vaccines against malaria. FEMS Microbiol Rev.

[7] Genton B, Betuela I, Felger I, Al-Yaman F, Anders R, Saul A (2002). A recombinant blood-stage malaria vaccine reduces *Plasmodium falciparum* density and exerts selective pressure on parasite populations in a phase 1-2b trial in Papua New Guinea. J Infect Dis.

[8] Healer J, Murphy V, Hodder A, Masciantonio R, Gemmill A, Anders R (2004). Allelic polymorphisms in apical membrane antigen-1 are responsible for evasion of antibody-mediated inhibition in *Plasmodium falciparum*. Mol Microbiol.

[9] Kiwanuka G (2009). Genetic diversity in *Plasmodium falciparum* merozoite surface protein 1 and 2 coding genes and its implications in malaria epidemiology: a review of published studies from 1997–2007. J Vector Borne Dis.

[10] Pattaradilokrat S, Sawaswong V, Simpalipan P, Kaewthamasorn M, Siripoon N, Harnyuttanakorn P (2016). Genetic diversity of the merozoite surface protein-3 gene in *Plasmodium falciparum* populations in Thailand. Malar J.

[11] Rebaudet S, Bogreau H, Silaï R, Lepere J, Bertaux L, Pradines B (2010). Genetic structure of *Plasmodium falciparum* and elimination of malaria, Comoros archipelago. Emerg Infect Dis.

[12] Papa Mze N, Ahouidi A, Diedhiou C, Silai R, Diallo M, Ndiaye D (2016). Distribution of *Plasmodium* species on the island of Grande Comore on the basis of DNA extracted from rapid diagnostic tests. Parasite.

[13] Atroosh W, Al-Mekhlafi H, Mahdy M, Saif-Ali R, Al-Mekhlafi A, Surin J (2011). Genetic diversity of *Plasmodium falciparum* isolates from Pahang, Malaysia based on MSP-1 and MSP-2 genes. Parasit Vectors.

[14] Zwetyenga J, Rogier C, Tall A, Fontenille D, Snounou G, Trape J (1998). No influence of age on infection complexity and allelic distribution in *Plasmodium falciparum* infections in Ndiop, a Senegalese village with seasonal, mesoendemic malaria. Am J Trop Med Hyg.

[15] Rich S, Ayala F (2000). Population structure and recent evolution of *Plasmodium falciparum*. Proc Natl Acad Sci USA.

[16] Kimura E, Mattei D, Mana di Santa S, Scherf A (1990). Genetic diversity in the major merozoite surface antigen of *Plasmodium falciparum* high prevalence of a third polymorphic form detected in strains derived from malaria patients. Gene.

[17] Kun F, Schmidt-Ott R, Lehman L, Lell B, Luckner D, Greve B (1998). Merozoite surface antigen 1 and 2 genotypes and rosetting of *Plasmodium falciparum* in severe and mild malaria in Lambarene, Gabon. Trans R Soc Trop Med Hyg.

[18] Snounou G, Zhu X, Siripoon N, Jarra W, Thaithong S, Brown KN (1999). Biased distribution of msp1 and msp2 allelic variants in *Plasmodium falciparum* populations in Thailand. Trans R Soc Trop Med Hyg.

[19] Kang J, Moon S, Kim J, Cho S, Lin K, Sohn W (2010). Genetic polymorphism of merozoite surface protein-1 and merozoite surface protein-2 in *Plasmodium falciparum* field isolates from Myanmar. Malar J.

[20] Zakeri S, Bereczky S, Naimi P, Pedro G, Djadid N, Färnert A (2005). Multiple genotypes of the merozoite surface proteins 1 and 2 in *Plasmodium falciparum* infections in a hypoendemic area in Iran. Trop Med Int Health.

[21] Ghanchi N, Matensson A, Ursing J, Jafri S, Bereczky S, Hussain R (2010). Genetic diversity among *Plasmodium falciparum* field isolates in Pakistan measured with PCR genotyping of the merozoite surface protein 1 and 2. Malar J.

[22] Gomez D, Chaparro J, Rubiano C, Rojas M, Wasserman M (2002). Genetic diversity of *Plasmodium falciparum* field samples from an isolated Colombian village. Am J Trop Med Hyg.

[23] Niang M, Loucoubar C, Sow A, Diagne M, Faye O, Faye O (2016). Genetic diversity of *Plasmodium falciparum* isolates from concurrent malaria and arbovirus co-infections in Kedougou, southeastern Senegal. Malar J.

[24] Niang M, Thiam L, Loucoubar C, Sow A, Sadio B, Diallo M (2017). Spatio-temporal analysis of the genetic diversity and complexity of *Plasmodium falciparum* infections in Kedougou, southeastern Senegal. Parasit Vectors.

[25] Ariey F, Chalvet W, Hommel D, Peneau C, Hulin A, Mercereau-Puijalon O (1999). *Plasmodium falciparum* parasites in French Guiana: limited genetic diversity and high selfing rate. Am J Trop Med Hyg.

[26] Takala S, Branch O, Escalante A, Kariuki S, Wootton J, Lal A (2002). Evidence for intragenic recombination in *Plasmodium falciparum*: identification of a novel allele family in block 2 of merozoite surface protein-1: asembo Bay Area Cohort Project XIV. Mol Biochem Parasitol.

[27] Chenet S, Branch O, Escalante A, Lucas C, Bacon D (2008). Genetic diversity of vaccine candidate antigens in *Plasmodium falciparum* isolates from the Amazon basin of Peru. Malar J.

[28] Conway D, McBride J (1991). Population genetics of *Plasmodium falciparum* within a malaria hyperendemic area. Parasitology.

[29] Basco L, Tahar R, Escalante A (2004). Molecular epidemiology of malaria in Cameroon. XVIII. Polymorphisms of the *Plasmodium falciparum* merozoite surface antigen-2 gene in isolates from symptomatic patients. Am J Trop Med Hyg.

[30] Ibara-Okabande R, Koukouikila-Koussounda F, Ndounga M, Vouvoungui J, Malonga V, Casimiro PN (2012). Reduction of multiplicity of infections but no change in msp2 genetic diversity in *Plasmodium falciparum* isolates from Congolese children after introduction of artemisinin-combination therapy. Malar J.

[31] Agyeman-Budu A, Brown C, Adjei G, Adams M, Dosoo D, Dery D (2013). Trends in multiplicity of *Plasmodium falciparum* infections among asymptomatic residents in the middle belt of Ghana. Malar J.

[32] Duah N, Matrevi S, Quashie N, Abuaku B, Koram K (2016). Genetic diversity of *Plasmodium falciparum* isolates from uncomplicated malaria cases in Ghana over a decade. Parasit Vectors.

[33] Mwingira K, Nkwengulila G, Schoepflin S, Sumari D, Beck H, Snounou G (2011). *Plasmodium falciparum msp1*, *msp2* and *glurp* allele frequency and diversity in sub-Saharan Africa. Malar J.

[34] Kidima W, Nkwengulila G (2015). *Plasmodium falciparum* msp2 genotypes and multiplicity of infections among children under 5 years with uncomplicated malaria in Kibaha, Tanzania. J Parasitol Res.

[35] Gosi P, Lanteri C, Tyner S, Se Y, Lon C, Spring M (2013). Evaluation pf parasite subpopulations and genetic diversity of *msp1*, *msp2* and *glurp* genes during and following artesunate monotherapy treatment of *Plasmodium falciparum* malaria in Western Cambodia. Malar J.

[36] Razak M, Sastu U, Norahmad N, Abdul-Karim A, Muhammad A, Munlandy P (2016). Genetic diversity of *Plasmodium falciparum* populations in malaria declining areas of Sabah, East Malaysia. PLoS One.

[37] Barry A, Schultz L, Senn N, Nale J, Kiniboro B, Siba P (2013). High levels of genetic diversity of *Plasmodium falciparum* populations in Papua New Guinea despite variable infection prevalence. Am J Trop Med Hyg.

[38] Congpuong K, Sukaram R, Prompan Y, Dornae A (2014). Genetic diversity of the msp-1, msp-2, and glurp genes of *Plasmodium falciparum* isolates along the Thai–Myanmar borders. Asian Pac J Trop Biomed.

[39] Issifou S, Rogier C, Adjagba-Olakpo M, Chabi-Worou N, Ntoumi F (2003). Complexity and genetic diversity of *Plasmodium falciparum* infections in young children living in urban areas of Central and West Africa. Parasitol Res.

[40] Njama-Meya D, Kamya M, Dorsey G (2004). Asymptomatic parasitemia as a risk factor for symptomatic malaria in a cohort of Ugandan children. Trop Med Int Health.

[41] Oyedeji S, Awobode H, Anumudu C, Kun J (2013). Genetic diversity of *Plasmodium falciparum* isolates from naturally infected children in north-central Nigeria using the merozoite surface protein-2 as molecular marker. Asian Pac J Trop Med.

[42] Soulama I, Nébié I, Ouédraogo A, Gansane A, Diarra A, Tiono A (2009). *Plasmodium falciparum* genotypes diversity in symptomatic malaria of children living in an urban and a rural setting in Burkina Faso. Malar J.

[43] Babiker H, Abdel-Muhsin A, Ranford-Cartwright L, Satti G, Walliker D (1998). Characteristics of *Plasmodium falciparum* parasites that survive the lengthy dry season in eastern Sudan where malaria transmission is markedly seasonal. Am J Trop Med Hyg.

[44] Paul R, Day K (1998). Mating patterns of *Plasmodium falciparum*. Parasitol Today.

[45] Legrand E, Volney B, Lavergne A, Tournegros C, Florent L, Accrombessi D (2005). Molecular analysis of two local falciparum malaria outbreaks on the French Guiana coast confirms the msp1 B-K1/varD genotype association with severe malaria. Malar J.

[46] Ekala M, Jouin H, Lekoulou F, Issifou S, Mercereau-Puijalon O, Ntoumi F (2002). *Plasmodium falciparum* merozoite surface protein 1 (MSP1): genotyping and humoral responses to allele-specific variants. Acta Trop.

[47] Babiker H, Walliker D (1997). Current views on the population structure of *Plasmodium falciparum*: implications for control. Parasitol Today.

[48] Branch O, Takala S, Kariuki S, Nahlen B, Kolczak M, Hawley W (2001). *Plasmodium falciparum* genotypes, low complexity of infection, and resistance to subsequent malaria in participants in the Asembo bay cohort project. Infect Immun.

[49] Anderson T, Williams J, Estrada-Franco J, Richardson L, Mollinedo R, Bockarie M (2000). Microsatellites reveal a spectrum of population structure in the malaria parasite *Plasmodium falciparum*. Mol Biol Evol.

[50] Anthony T, Cox-Singh J, Matusop A, Ratnam S, Shamsul S, Singh B (2005). Fragmented population structure of *Plasmodium falciparum* in a region of declining endemicity. J Infect Dis.

[51] Ebrahimzadeh A, Mohammadi S, Jamshidi A (2014). Allelic forms of merozoite surface protein-3 in *Plasmodium falciparum* isolates from southeast of Iran, Jundishapur. J Microbiol.

[52] Soulama I, Bigoga J, Ndiaye M, Bougouma E, Quagraine J, Casimiro P (2011). Genetic diversity of polymorphic vaccine candidate antigens (apical membrane antigen-1, merozoite surface protein-3, and erythrocyte binding antigen-175) in *Plasmodium falciparum* isolates from western and central Africa. Am J Trop Med Hyg.

[53] Gardner M, Hall N, Fung E, White O, Berriman M, Hyman R (2002). Genome sequence of the human malaria parasite *Plasmodium falciparum*. Nature.

[54] Jordan S, Oliveira A, Hernandez J, Oster R, Chattopadhyay D, Branch O (2011). Malaria immunoepidemiology in low transmission: correlation of infecting genotype and immune response to domains of *Plasmodium falciparum* merozoite surface protein 3. Infect Immun.

